# Discriminative validity and diagnostic accuracy of Horus® posturographic parameters in individuals with and without vestibulopathy

**DOI:** 10.1016/j.bjorl.2025.101746

**Published:** 2026-01-09

**Authors:** Maria Clara Peixoto Marinheiro, Ana Clara Teixeira Fernandes, Luana Dantas da Silva, Adriana Guedes Carlos, José Diniz Júnior, Juliana Maria Gazzola, Vanessa Regiane Resqueti

**Affiliations:** a Federal University of Rio Grande do Norte (UFRN), Department of Physiotherapy, Postgraduate Program in Physiotherapy, Natal, RN, Brazil; b Federal University of Rio Grande do Norte (UFRN), Department of Surgery, Postgraduate Program in Otorhinolaryngology, Natal, RN, Brazil; c Federal University of Rio Grande do Norte (UFRN), Onofre Lopes University Hospital (HUOL), Pneumocardiovascular Lab, Department of Physiotherapy, Natal, RN, Brazil

**Keywords:** Vestibular diseases, Postural balance, Dizziness, Psychometrics

## Abstract

**Objective:**

To assess the discriminative validity and diagnostic accuracy of posturographic parameters from the Horus® in individuals with and without vestibulopathy.

**Methods:**

This is a methodological study of discriminative validity and diagnostic accuracy. 153 individuals aged 40–79, of both sexes, were included and divided into Group 1 (with vestibular dysfunction) and Group 2 (without vestibular dysfunction). Instruments on clinical characterization, cognition, physical activity level, and posturography were applied. Discriminative validity was assessed with nonparametric multivariate analyses, the Mann-Whitney test, controlled with Holm adjustment and rank-biserial correlation to estimate effect size. Sensitivity, specificity, area under the curve, and cutoff points were calculated using ROC curves.

**Results:**

The final sample included 78 individuals in Group 1 and 75 in Group 2, with mean ages of 59.36 and 56.48 years, respectively. Significant differences were found between the groups for all dependent variables related to confidence ellipse/limit of stability and mediolateral velocity (p < 0.001), with moderate to large differences (ES > 0.28) and in most of the anteroposterior velocity and sensory analysis (p < 0.005), with small to moderate differences (ES: >0.11 and <0.43). The ROC curve analysis revealed that confidence ellipse/limit of stability and mediolateral velocity exhibited high specificity (84% and 80%) and moderate sensitivity (52.6% and 53.85%). The Vestibular Function parameter demonstrated good sensitivity (70%) and moderate specificity (60.8%), while the Composite Balance Index showed a sensitivity of 79.5% and specificity of 64%.

**Conclusion:**

Horus® posturographic parameters demonstrated moderate to good discriminative ability to differentiate individuals with and without vestibular dysfunction, with high sensitivity for vestibular function and the Composite Balance Index.

**Level of evidence:**

3 (cross-sectional study).

## Highlights

• CE/SL and MV/ML in C4 are effective for ruling out vestibular dysfunction.
• Vestibular Function and CBI show high sensitivity in vestibular disorder diagnosis.
• Moderate to large differences were observed for Visual Function, Vestibular, and CBI.
• Horus® parameters enhance vestibular dysfunction diagnosis and rehabilitation.
• Posturographic parameters showed moderate-to-good discriminative validity.

## Introduction

The coordinated action of the vestibular, somatosensory, and visual systems together with the Central Nervous System allows good performance in accurate responses to situations that disrupt Postural Balance (PB).^1^^,^^2^ In the absence or reduction of somatosensory and visual information, there is greater activation of the Vestibular System (VS), which is sensitive to head movement and coordinates eye motricity with the head, postural adjustment, and gait.^3^ Peripheral and/or central alterations in the VS are accompanied by dizziness, vertigo, vestibule-visual, and postural symptoms, which become more evident with increased age^4^, ^5^, ^6^ and can cause falls and fractures, functional limitations, and restrictions in social participation.^7^^,^^8^

The assessment of individuals with Vestibular Dysfunction (VD) is based on clinical history and instruments that evaluate vestibular function and postural stability, such as functional and posturographic tests.^9^^,^^10^ Horus® posturography, created in Brazil, allows the integration of clinical, kinetic-functional diagnosis and rehabilitation of people with VD.^11^^,^^12^

The “Consensus-based Standards for the Selection of Health Measurement Instruments (COSMIN)” for discriminative validity, through a hypothesis test, demonstrates the ability of an instrument to discriminate groups with distinct health conditions, and the “Standards for Reporting Diagnostic Accuracy Studies (STARD)” aim to improve the quality of diagnostic accuracy research to increase the safety of the findings.^13^^,^^14^

Few methodological studies examine the discriminative validity and accuracy of static posturography to discriminate individuals with and without VD and identify cutoff points for Brazilians. In this sense, this study aims to evaluate the discriminative validity and diagnostic accuracy of Horus® posturographic parameters in seven sensory conditions to discriminate individuals with and without VD.

## Methods

The methodological research on discriminative validity and diagnostic accuracy was conducted between March 2023 and March 2025 at the Department of Physiotherapy of Universidade Federal do Rio Grande do Norte (UFRN). It was based on the COSMIN and STARD consensuses,^13^^,^^14^ approved by the Research Ethics Committee of the UFRN under opinion number 6.810.274, and it followed the guidelines of the Declaration of Helsinki for studies involving human subjects. All volunteers read and signed the Free and Informed Consent Form.

The sample consisted of individuals aged 40–79, of both sexes, distributed into two groups:

Group 1 (G1): Consisted of individuals diagnosed with VD, postural imbalance, dizziness, and/or vertigo, recruited from the Otoneurology Outpatient Clinic of a University Hospital in the city of Natal/RN. The clinical diagnosis was based on anamnesis, physical examination, complementary tests (VHIT, tone audiometry, vocal, immittancemetry), imaging, and laboratory tests.

Group 2 (G2): consisted of individuals without a diagnosis of VD, postural imbalance, dizziness, or vertigo, recruited by convenience sampling. Participants were assessed through a targeted clinical anamnesis and subjected to exclusion criteria based on the protocol published by Nishino et al. (2021),^12^ which included a history of migraine, motion sickness, and the use of antivertiginous medications.

Participants were required to have good visual acuity, assessed by the Snellen Optometric Chart at a distance of three meters, and needed to read, with glasses and/or lenses, from top to bottom, up to the third line.^15^

Individuals were excluded due to cognitive alteration by the Leganés Cognitive Test (PCL^16^; self-report pain and alteration in lower limbs that interfere with strength and mobility; neurological symptoms with motor compromise^17^; weight over 130 kg^12^ and those who do not understand the assessor's instructions.

In an interview, sociodemographic (sex and age group) and clinical-functional data were collected: height, in meters (m), weight in kilograms (kg), and Body Mass Index (BMI) (kg/m^2^). Individuals were classified according to BMI based on the criteria for elderly people^18^ and adults.^19^ Also, questions were asked about the syndromic and topographic diagnosis of VD, the vestibular affection, symptom onset time, type, and intensity in the last 24 h on the Numeric Symptom Scale, for 0 “no dizziness” and 10 “intense dizziness”.^20^^,^^21^

The PCL was applied for cognitive screening, a questionnaire validated for the Brazilian older adult population, applicable to different school levels, and with a cutoff for cognitive deficit of 22 points.^16^

The International Physical Activity Questionnaire (IPAQ) assessed the level of physical activity based on the time spent active in the last week in 10 continuous minutes. The individuals were classified as active and very active, irregularly active, or sedentary.^22^

Two trained researchers performed the Static Posturography with Dynamic Tests Horus® in a lit room. The Horus® system consists of a force platform, four sensors, and software that provides different visual and proprioceptive stimuli indicated for the assessment and rehabilitation of PB. Also, it requires a computer and television to connect to the platform.^11^^,^^12^

The tests were performed on a stable and unstable surface, the latter created by a cushion that reduces somatosensory information and recreates everyday situations.^11^^,^^12^ During the assessment, the volunteer was instructed on positioning, sequences, and condition interruptions, according to Fernandes et al.^23^

The software generated the area of the Stability Limit (SL), 95% Confidence Ellipse (CE) (area containing 95% of the Center of Pressure [COP] points during the test – mm^2^), Mean Velocity (MV) total (total length of the COP over the base of support divided by test time - mm/s), and the MV in Anteroposterior (AP) and Mediolateral (ML) directions.^11^^,^^12^

Furthermore, the CE/SL ratio indicates greater postural stability as its score decreases. The Residual Functional Balance (RFB%) assesses PB performance based on the available residual area for safe oscillation, where values close to 100% reflect greater PB.^11^^,^^12^

The Sensory Analysis (SA) (Somatosensory Function (SOM%), Visual Function (VIS%), Vestibular Function (VEST%), Right Visual Dependence (VDepR%), Left Visual Dependence (VDepL%), and Tunnel Visual Dependence (VDepT%)) analyzes the contribution of systems to maintaining PB. The Composite Balance Index (CBI) indicates the overall coordination of PB.^11^^,^^12^ SA results below the reference may indicate hypofunction of the sensory system. The formulas for each parameter can be observed in Barboza and Tavares.^11^

The sample size was considered based on COSMIN,^13^ which establishes a minimum of 50 to 99 individuals per group. The variables were analyzed using the Statistical Package for the Social Sciences (SPSS version 20.0, IBM, New York, USA), with a significance level of p ≤ 0.05. The Kolmogorov-Smirnov test was applied to verify data normality, followed by descriptive analysis.

To investigate significant differences between the variables and groups, non-parametric multivariate analyses (MANOVA) were performed using the MANOVA.RM package in the *R* language (version 4.3.0, R Core Team, 2023) with the Modified ANOVA-Type Statistic (MATS).^24^ The Mann–Whitney test was applied to assess individual comparisons of CE/SL, MV/ML, MV/AP, and SA between groups with and without VD, followed by Holm’s adjustment (1979).^25^

Subsequently, Effect Size (ES) was determined using rank-biserial correlation, where values ≥ 0.11 indicate a small effect, ≥0.28 a medium effect, and ≥0.43 a large effect.^26^ For this, the Jamovi software (The Jamovi Project, 2024^27^ was used. Additionally, discriminative validity was only accepted when 75% of the hypotheses were met.^28^

The cutoff point for the variables CE/SL, MV/ML, and MV/AP in C4, Vestibular Function, and CBI was obtained using the Receiver Operating Characteristic (ROC) curve to identify groups with and without VD. Furthermore, the Area Under the Curve (AUC), 95% Confidence Interval (95% CI), and the sensitivity and specificity values were calculated, with the AUC being closer to 1.0 indicating better discriminatory ability.^14^

## Results

A total of 160 individuals were recruited, with four from G1 and three from G2 excluded due to musculoskeletal limitations and neurological diseases, totaling 153 individuals (78 in G1 and 75 in G2). Table 1, Table 2 present the sample characterization, and no statistically significant differences were found between the groups for age range (p = 0.096) and sex (p = 0.201).

**Table 1:** Sociodemographic and clinical-functional characterization of individuals with (n = 78) and without vestibular dysfunction (n = 75).

		With VD (n = 78)	Without VD (n = 75)
Age range	40 to 59 years	39 (50.0%)	46 (61.3%)
	60 to 69 years	22 (28.2%)	22 (29.3%)
	70 to 79 years	17 (21.8%)	7 (9.3%)
Sex	Male	15 (19.2%)	21 (28.0%)
	Female	63 (80.8%)	54 (72.0%)
BMI Classification (kg/m^2^)	<22 (underweight)	1 (1.3%)	2 (2.7%)
	22.1 a 26.9 (Eutrophic)	34 (43.6%)	40 (53.3%)
	27 = Overweight	34 (43.6%)	25 (33.6%)
Low physical activity levels (IPAQ)	>30 = Obesity	9 (11.5%)	8 (10.7%)
	Yes	31 (39.7%)	20 (26.7%)
	No	47 (60.3%)	55 (73.3%)

n, absolute frequency; %, Relative frequency; BMI (kg/m^2^), Body Mass Index; IPAQ, International Physical Activity Questionnaire.

**Table 2:** Characterization of symptoms and diagnosis of vestibular disorders in individuals with vestibular dysfunction (n = 78).

Clinical Characteristics	Category	n (%)
Symptom Onset Time	0 to 1 year	12 (15.6%)
	1 to 4 years	27 (35.1)
	More than 5-years	38 (49.4%)
Type	Vertigo	23 (29.9%)
	Dizziness	14 (18.2%)
	Both	40 (51.9%)
Syndromic and Topographic Diagnosis	Unilateral Peripheral Syndrome	60 (39.2%)
	Bilateral Peripheral Syndrome	2 (1.3%)
	Central Syndrome	10 (6.5%)
	Mixed Syndrome	6 (3.9%)
**Peripheral Etiologies**		
Benign Paroxysmal Positional Vertigo		43 (28.1%)
Menière’s Disease		4 (2.6%)
Unilateral Vestibulopathy After Medical Procedure		4 (2.6%)
Others		10 (6.6%)
**Central Etiologies**		
Vestibular Migraine		6 (3.9%)
Persistent Postural-Perceptual Dizziness		8 (5.2%)
Vertebrobasilar Insufficiency		4 (2.7%)
**Other Associated Syndromes**		
Metabolic Labyrinthopathies		12 (7.8%)

n, Absolute frequency; %, Relative frequency.

There was a statistically significant multivariate effect for the dependent variables CE/SL, MV/ML, MV/AP, and AS between groups (MATS = 75.98; 122.28; 45.66; 33.15; p ≤ 0.01), respectively.

Individual analyses indicated statistically significant differences between groups, with a moderate to large effect for CE/SL and MV/ML in all conditions (p < 0.001) and a small to moderate effect for MV/AP in most variables, except for “Optokinetic to the right on unstable surface” (Table 3). Significant differences ranging from moderate to large were observed for Visual Function, Vestibular Function, and CBI (Table 4).

**Table 3:** Comparison of CE/SL (Confidence Ellipse/Limit of Stability), MV/ML (Mean Velocity Mediolateral), and MV/AP (Mean Velocity Anteroposterior) in sensory conditions between individuals with (n = 78) and without vestibular dysfunction (n = 75).

	Conditions	Groups	Median	IQR_25%‒75%_	p	ES
**CE/LS**	EO/SS	G1	1.35	0.70‒2.82	**<0.001**	0.41
		G2	0.60	0.40‒1.00		
	EC/SS	G1	2.45	1.00‒6.00	**<0.001**	0.46
		G2	0.80	0.50‒1.40		
	EO/US	G1	5.05	2.60‒9.17	**<0.001**	0.42
		G2	2.70	1.80‒4.20		
	EC/US	G1	10.55	5.37‒19.60	**<0.001**	0.45
		G2	4.90	3.50‒7.50		
	OR/US	G1	5.65	3.47‒12.05	**<0.001**	0.35
		G2	3.50	2.40‒6.40		
	OL/US	G1	6.30	3.85‒12.85	**<0.001**	0.38
		G2	3.90	2.50‒5.60		
	OT/US	G1	6.30	3.77‒11.30	**<0.001**	0.51
		G2	3.00	1.60‒4.20		
**MV/ML**	EO/SS	G1	4.75	3.70‒7.80	**<0.001**	0.36
		G2	3.80	3.00‒4.90		
	EC/SS	G1	6.60	4.77‒10.92	**<0.001**	0.41
		G2	4.90	3.60‒6.20		
	EO/US	G1	12.50	9.90‒16.17	**<0.001**	0.42
		G2	9.50	8.00‒11.80		
	EC/US	G1	20.30	15.60‒25.75	**<0.001**	0.42
		G2	14.00	11.50‒19.10		
	OR/US	G1	14.75	11.15‒19.42	**<0.001**	0.38
		G2	11.40	9.00‒14.40		
	OL/US	G1	15.30	10.72‒19.20	**<0.001**	0.41
		G2	11.20	8.90‒14.30		
	OT/US	G1	12.75	10.37‒16.62	**<0.001**	0.49
		G2	8.80	7.70‒11.20		
**MV/AP**	EO/SS	G1	8.45	6.50‒11.22	**<0.001**	0.34
		G2	6.60	5.80‒8.20		
	EC/SS	G1	12.95	8.55‒17.72	**0.002**	0.30
		G2	9.60	7.30‒12.60		
	EO/US	G1	14.45	11.97‒20.10	**0.013**	0.23
		G2	13.60	10.70‒16.00		
	EC/US	G1	25.35	20.65‒36.20	**0.043**	0.19
		G2	24.10	19.00‒29.20		
	OR/US	G1	18.65	15.02‒23.80	0.082	0.16
		G2	16.90	13.90‒20.80		
	OR/US	G1	19.55	15.75‒25.05	**0.036**	0.20
		G2	17.60	13.60‒20.60		
	OT/US	G1	17.95	14.12‒22.37	**0.003**	0.28
		G2	15.00	12.70‒19.40		

CE/SL, Confidence Ellipse/Limit of Stability; MV/ML, Mean Velocity Mediolateral; MV/AP, Mean Velocity Anteroposterior; EO, Eyes Open; EC, Eyes Closed; OR and OL, Optokinetic to the Right and Left; OT, Optokinetic Tunnel; SS, Stable Surface; US, Unstable Surface; G1, With vestibular dysfunction; G2, Without vestibular dysfunction; IQR, Interquartile Range; ES, Effect Size (rank-biserial correlation).

**Table 4:** Comparison of sensory analyses between individuals with (n = 78) and without vestibular dysfunction (n = 75).

Sensory Analysis	Group	Median	IQR_25%‒75%_	p	ES
Somatosensory function	G1	99.40	97.07‒100.00	**0.01**	**0.24**
	G2	99.70	99.50‒100.00		
Visual function	G1	96.30	93.60‒98.50	**<0.001**	**0.32**
	G2	97.90	96.90‒98.80		
Vestibular function	G1	91.30	82.30‒96.00	**<0.001**	**0.40**
	G2	96.00	93.60‒97.20		
Right visual dependence	G1	102.30	99.67‒108.32	**0.02**	**0.21**
	G2	101.00	100.10‒103.10		
Left visual dependence	G1	102.40	99.42‒107.10	0.11	0.15
	G2	100.70	99.90‒102.70		
Optokinetic tunnel dependence	G1	102.15	97.75‒107.22	0.77	0.03
	G2	101.90	100.70‒103.70		
Compositive Equillibrium Index	G1	92.85	86.50‒95.80	**<0.001**	**0.52**
	G2	96.70	95.00‒97.60		

G1, With vestibular dysfunction; G2, Without vestibular dysfunction.

**Fig. 1:**
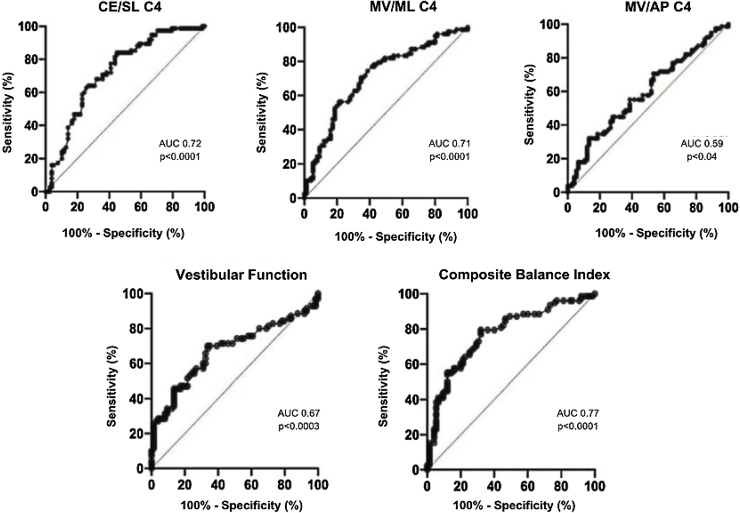
ROC curves and sensitivity and specificity values for CE/SL, MV/ML, MV/AP, Vestibular Function, and CBI. CE/SL, Confidence Ellipse/Limit of Stability; MV/ML, Mean Velocity Mediolateral; MV/AP, Mean Velocity Anteroposterior; CBI, Compositive Balance Index; AUC, Area Under the Curve. Source: Authors (2024).

The AUC of the variables CE/SL, MV/ML, MV/AP, Vestibular Function, and CIB from C4 are represented in Fig. 1. The cutoff points to identify individuals with and without VD, along with sensitivity and specificity, were, respectively: > 10.05 (52.56%, 84.00%), > 19.65 (53.85%, 80%), > 22.85 (67.95%, 46.67%), < 95.5 (70%, 60.81%), < 96.15 (79.5%, 64%).

## Discussion

This study analyzed the discriminative validity and diagnostic accuracy of Horus® posturography parameters in distinguishing individuals with and without Vestibular Dysfunction (VD). Our findings demonstrated that Horus® posturography exhibited moderate discriminative capacity in differentiating individuals with and without VD, with CE/SL and VM/ML in C4 being the most effective parameters for ruling out the dysfunction. Although no studies have specifically evaluated the discriminative validity of these parameters in differentiating these groups, our results align with previous research that establishes distinctions in postural sway between groups, particularly in CE 95% area, MV/ML, AP, and sensory analysis.^29^^,^^30^ Furthermore, studies comparing Postural Balance (PB) between individuals with and without VD have reported significant differences in Center of Pressure (CP) area and VM, especially under conditions of somatosensory and visual deprivation.^31^^,^^32^ CE/SL and MV/ML differed between groups in all conditions, with a moderate to large Effect Size (ES) and more significant postural instabilities in the VD group, mainly under conditions that impose greater demand on the VS, such as C4, which reduces visual and somatosensory feedback, and situations involving visual distortions. These findings agree with studies that used different platforms, such as the Balance Rehabilitation Unit (BRU^31^ and the Tetrax IBSTM, in individuals with VD.^14^^,^^32^, ^33^, ^34^

In MV/AP, individuals with VD exhibited greater values for this parameter across all conditions due to difficulties in sensory integration to maintain stability.^5^^,^^35^^,^^36^ However, the lower ES observed may be explained by the adoption of compensatory postural strategies in the VD group, such as increased muscle co-contraction^37^ and individual variability.

It has been established that individuals with VD exhibit lower performance when exposed to visual conflicts,^14^^,^^30^^,^^31^^,^^33^ as expected in the initial hypotheses and confirmed in the present study, except for MV/AP parameters in rightward optokinetic, VDepR%, VDepL%, and VDepT%. This may be justified by the probable compensatory abilities of the VD group,^38^ particularly in directional stimuli, due to vestibular asymmetries, individual variability, and sample-specific characteristics. Repeated exposure to complex visual stimuli in daily life, such as environments with conflicting, may lead to postural adjustments and increased muscle activation,^38^ in addition to the influence of the visual field and externally instructed attentional focus.^39^ Furthermore, it is important to note that VDepR%, VDepL%, and VDepT% consider the RFB%, which corresponds to the area still available for safe postural sway.^11^

Vestibular function, visual function, and the CBI showed moderate to large differences between groups. However, for the SOM, a small effect size was observed. These results are consistent with previous studies demonstrating lower scores in somatosensory, visual, vestibular, and CBI analyses in individuals suffering from dizziness and experiencing recurrent falls.^29^^,^^30^^,^^40^ Nevertheless, individuals with VD may develop somatosensory compensatory strategies to compensate for vestibular hypofunction,^36^ which may explain the smaller differences observed.

Therefore, high specificities were found for CE/SL (84%) and MV/ML (80%) in C4, however, sensitivities were limited (52.56%, 53.85%). C4 is the most challenging condition, as the individual relies primarily on the VS.^30^ Thus, due to its clinical plausibility, the accuracy of this condition was evaluated. Our results highlighted the usefulness of these parameters in ruling out the presence of VD and corroborated studies that have identified high specificities (>90%) in posturography.^41^ Furthermore, similarities were observed between the sensitivity of static posturography (53%)^41^ and our findings, reinforcing the need to complement posturography with other vestibular tests.^41^

On the other hand, the highest sensitivity observed for Vestibular Function (70%) may be explained by its relationship with the EFR% in C4, where the individual fundamentally depends on the vestibular system to maintain postural control, and with the baseline condition, in which all sensory inputs are available.^11^ Our findings support the diagnostic accuracy of both dynamic and static posturography with head movement in patients with unilateral vestibular hypofunction, with greater sensitivity in C4.^42^^,^^43^

The CBI reflects the integration between sensory systems and overall postural control coordination.^11^ This parameter demonstrated good sensitivity (79.5%) due to its ability to detect sensory integration deficits in individuals with VD, as observed in other studies.^30^ However, its interpretation should be cautious, as it considers not only the vestibular system but also the somatosensory and visual systems in isolation.^11^

Sociodemographic findings indicate a predominance of women, which aligns with evidence suggesting that hormonal changes compromise labyrinthine homeostasis and lead to vestibular symptoms, especially in middle-aged and older individuals.^44^^,^^45^ This occurs due to the reduction of hair cells and bone demineralization associated with aging, which increases the risk of BPPV, a frequent etiology confirmed in our sample.^36^^,^^44^, ^45^, ^46^ Clinical results also demonstrated a higher BMI and lower levels of physical activity in individuals with VD, which supports previous reviews associating overweight with VD and activity restrictions due to motion-induced symptoms in this population.^36^^,^^46^^,^^47^

Furthermore, it is crucial to note that, although rigorous and scientifically based exclusion criteria were applied to confirm the absence of VD in the control group, the lack of diagnostic tests, such as vHIT, Vestibular Evoked Myogenic Potentials (VEMP), and caloric testing, may have influenced the accuracy of group classification and, consequently, the sensitivity and specificity of the analyzed parameters. This limitation may have resulted in equivalent posturographic patterns among individuals, thereby reducing the discriminative capacity of certain variables. In this context, future studies are encouraged to combine formal vestibular tests with clinical criteria to ensure greater confidence in group categorization and, consequently, improve the diagnostic accuracy of the tool.

Similarly, although methodological challenges exist, it is essential to establish the advantages of using the Horus® posturography system in comparison with other tools, such as the BRU,^31^ Tetrax (IBSTM),^34^ and EquiTest® (NeuroCom).^48^ The platform used in the present study is nationally manufactured and offers substantial benefits in terms of cost and accessibility, as it requires lower financial investment and infrastructure. Moreover, when comparing previous research involving other posturography systems, a variety of stimuli and protocols have been employed. As a result, direct comparison of clinical outcomes across different platforms is limited due to variations in the assessment methods of postural sway. Nevertheless, Horus® was shown to be capable of identifying differences between groups with and without Vestibular Dysfunction (VD), demonstrating consistency with previously reported findings using other established instruments.^31^^,^^34^ This reinforces the tool’s utility in assessing individuals with VD.

Moreover, given the observed results, it is essential to consider that the Horus posturography system should not be regarded as a definitive diagnostic tool, but rather as a promising complementary instrument in the clinical and functional assessment of individuals with vestibular disorders, to be used in conjunction with other otoneurological examinations.

The study's limitations include the sample size, which is a critical factor in diagnostic accuracy studies, and the absence of cutoff points for different age groups. Therefore, future studies should include larger samples, and age subgroups to define more precise cutoff points and methodologies incorporating head movement in the posturographic model. Nevertheless, it is significant to highlight the use of a national, high-tech, and cost-effective instrument compared to international alternatives, as well as the incorporation of dynamic tests such as LE and the application of methodologically rigorous checklists.

## Conclusion

The Horus® posturography demonstrated moderate discriminative capacity in differentiating individuals with and without VD, with EC/LE and MV/ML in C4 being the most effective parameters for ruling out dysfunction. Conversely, Vestibular Function and CBI exhibited greater sensitivity. These results emphasize the applicability of Horus® in distinguishing individuals with and without VD, aiming to support precise clinical and functional diagnosis, facilitate targeted vestibular rehabilitation programs, and assist in monitoring treatment progression in these patients.

## ORCID ID

Maria Clara Peixoto Marinheiro: 0000-0002-1732-156X

Ana Clara Teixeira Fernandes: 0000-0001-6672-3736

Luana Dantas da Silva: 0000-0001-9969-4601

Adriana Guedes Carlos: 0000-0003-0747-5044

José Diniz Júnior: 0000-0002-2327-945X

Juliana Maria Gazzola: 0000-0002-9333-1831

Vanessa Regiane Resqueti: 0000-0003-4817-9364

## Declaration

During the preparation of this work, the authors used ChatGPT to assist in identifying spelling and writing errors. After using this tool, the authors reviewed and edited the content as necessary and assume full responsibility for the content of the publication.

## Funding

This study was supported by a CAPES (Coordination for the Improvement of Higher Education Personnel) scholarship.

## Data availability statement

We declare that all data are available in repository.

## Declaration of competing interest

The authors declare no conflicts of interest.
